# Survival Benefit of Pyloric Lymph Node Dissection for Siewert Type II/III Adenocarcinoma of the Esophagogastric Junction Based on Tumor Diameter: A Large Cohort Study

**DOI:** 10.3389/fonc.2021.748694

**Published:** 2021-12-01

**Authors:** Xia Lin, Zhengyan Li, Chenjun Tan, Xiaoshuang Ye, Jie Xiong, Jiajia Liu, Ao Mo, Yan Shi, Feng Qian, Peiwu Yu, Yongliang Zhao

**Affiliations:** ^1^ Department of General Surgery, The First Affiliated Hospital of Army Medical University, Chongqing, China; ^2^ Department of Gastrointestinal Surgery, Three Gorges Hospital, Chongqing University, Chongqing, China

**Keywords:** gastric cancer, AEG, Siewert classification, lymphadenectomy, prognosis

## Abstract

**Background:**

It is unclear whether the dissection of pyloric lymph nodes (PLNs, No. 5 and No. 6 lymph nodes) is necessary for adenocarcinoma of the esophagogastric junction (AEG) with a tumor diameter >4 cm based on current guidelines. This study aimed at evaluating whether pyloric node lymphadenectomy is essential for patients with Siewert type II/III AEG according to different tumor diameters.

**Methods:**

This study included 300 patients on whom transabdominal total gastrectomy was performed for Siewert type II/III AEG at a high-volume center in China from January 2006 to December 2015. The index of estimated benefit from lymph node dissection (IEBLD) was used to analyze the priority of pyloric lymphadenectomy.

**Results:**

In Siewert type II AEG, the 5-year overall survival (OS) and the 5-year disease-free survival (DFS) were similar between patients with PLN-positive cancer and patients of stage III AEG without PLN metastasis (23.1% vs. 30.6%, *p* = 0.505; 23.1% vs. 27.1%, *p* = 0.678). However, in Siewert type III AEG, the OS and the DFS of patients with PLN-positive cancer were significantly lower than that of patients with stage III without PLN metastasis (7.9% vs. 27.8%, *p* = 0.021; 0 vs. 26.8%, *p* = 0.005). According to the IEBLD, the dissection of PLNs did not appear to be beneficial in either Siewert type II AEG or type III AEG, whereas a stratified analysis revealed that PLN dissection yielded a high therapeutic benefit for Siewert type II AEG with tumor diameters >4 cm.

**Conclusion:**

We recommended that the PLNs be dissected in Siewert type II AEG when a tumor diameter is >4 cm. Total gastrectomy should be optional for Siewert type II AEG with a tumor diameter >4 cm and Siewert type III AEG.

## Introduction

The increasing frequency of adenocarcinoma of the esophagogastric junction (AEG) has raised concern worldwide (1). This trend may be due to the elimination of *Helicobacter pylori* and the increase in reflux gastroesophagitis. Based on the anatomic location of the tumor epicenter, AEG is classified into three subgroups (types I, II, and III) (2). Siewert type II and type III AEGs are more common in Eastern countries, which are mainly characterized by abdominal lymph node metastasis, but there are also differences in lymph node metastasis since they are different subtypes with different biological characteristics (3). Therefore, Siewert type III AEGs are often considered as gastric cancer with a transhiatal approach (4). Although the surgical approach for Siewert type II AEG remains controversial, clinicians usually recommend gastrectomy with resection of the lower esophagus for Siewert type II AEG (5). Radical gastrectomy with regional lymphadenectomy is the primary surgical strategy for AEG (6). The status of lymph node metastasis in AEG with a tumor diameter ≤4 cm was evaluated by the Japanese Gastric Cancer Association (JGCA), which also developed a flow diagram to identify the extent of the lymphadenectomy. The pyloric lymph nodes (PLNs, No. 5 and No. 6 lymph nodes) was not recommended to be dissected in the 5th edition of the Japanese gastric cancer treatment guidelines because the incidence of PLN metastasis in AEG with tumor diameter ≤4 cm was less than 1% (7, 8). However, the necessity of PLN lymphadenectomy was not mentioned for AEG with a tumor diameter >4 cm. Additionally, several studies from China demonstrated that the incidence of PLN metastasis exceeded 10% in AEG (9, 10). Recently, a prospective nationwide multicenter study revealed that the metastasis rate of PLNs was more than 10% in tumors >6 cm (11). Therefore, the incidence of PLN metastasis is controversial. In China, over 80% of the AEG patients are diagnosed at advanced stages and AEG cases in which the tumor diameter is >4 cm account for a large proportion (12, 13). To date, no consensus has been established on the extent of lymph node dissection for AEG with tumor >4 cm. This study aimed at evaluating the survival benefits of PLN dissection for Siewert type II/III AEG with tumor diameter ≤4 and >4 cm.

## Patients and Methods

### Patients

Radical total gastrectomy with transhiatal approach was performed on 424 consecutive patients with Siewert type II/III AEG at the Department of General Surgery, Southwest Hospital of Army Medical University from January 2006 to December 2015. The surgically resected specimens were assessed for classification of Siewert type by experienced gastrointestinal pathologist and surgeon according to the length and location of the tumor and results of gastroscopy, upper gastrointestinal barium meal, and abdominal computed tomography (14). The guideline defined the esophagogastric junction (EGJ) as a lower margin of palisading small vessels on gastroscopy (15). The Siewert type II AEG was defined as the center of the tumor located from 2 cm below to 1 cm above the EGJ, and type III was 2 to 5 cm below the EGJ (2). The included patients had pathologically confirmed Siewert type II/III AEG, radical total gastrectomy (R0 resection) with resection of ≥16 lymph nodes, underwent a transabdominal approach, esophageal invasion less than 3 cm, and showed no evidence of distant metastasis. Patients were excluded if they had remnant gastric cancer, had distant metastasis or peritoneal dissemination, and underwent R1 or R2 resection. Our study aimed to investigate the distribution of lymph node metastasis for Siewert type II and III AEG. To accurately assess the pathological status of each lymph node after surgery, patients who received neoadjuvant chemotherapy were excluded from this study. Ultimately, 300 patients were included in the final analyses.

Our study was approved by the Ethics Committee of Southwest Hospital, Army Medical University (No. KY2020272).

### Clinical Parameters

Sex, age, body mass index (BMI), physical status according to the American Society of Anesthesiologists (ASA) criteria, tumor size, histological type, surgical procedure, depth of tumor invasion, nodal stage, tumor-node-metastasis (TNM) stage, number of lymph nodes retrieved, number of metastatic lymph nodes, and adjuvant chemotherapy were included as clinicopathological features. The pathological tumor stage was assessed according to the 8th edition of the Union for International Cancer Control/AJCC TNM classification (16). Siewert types II and III AEGs were staged by TNM esophageal classification and TNM gastric classification, respectively. In this study, the No. 5 and No. 6 lymph nodes were defined as PLNs (17). Tumor histology was assessed according to the 14th edition of the classification (18). Well-differentiated and moderately differentiated tubular adenocarcinoma and papillary adenocarcinoma were classified as differentiated tumors. Undifferentiated tumors included poorly differentiated adenocarcinoma, signet-ring cell carcinoma, and mucinous carcinoma.

### Follow-Up and Long-Term Outcomes

The primary outcomes were 5-year overall survival (OS) and 5-year disease-free survival (DFS). A minimum follow-up period of 5 years was required in our study. Six to eight cycles of postoperative chemotherapy with fluorouracil and platinum were recommended for patients with advanced AEG or lymph-node metastasis in any T stage. All patients were followed up every month for the first 2 years and then every 6 months for the next 3 years. Our last follow-up was conducted by telephone through outpatient department visits or home visits in December 2020. The OS was defined as the day of gastrectomy to the date of death or last follow-up. The DFS was defined as the interval from surgery to first recurrence or death.

### Evaluation of the Therapeutic Value of Intraabdominal Lymph Node Dissection

In this study, the index of estimated benefit from lymph node dissection (IEBLD) was used to evaluate the priority of nodal dissection introduced by Sasako et al. in 1995 (19). The calculation of index was defined as multiplying the frequency of metastasis to the station by the 5-year survival rate of patients with metastasis to that station (20). The 5-year survival rate of patients with lymph node metastasis was calculated for each nodal station independently, irrespective of metastasis to other lymph node stations (20). The priority of nodal dissection was assessed by the value of the index at each station that had an IEBLD above 3.0 (21).

### Statistical Analyses

We used the Chi-square test or Fisher’s exact test to compare categorical variables and Student’s *t*-test to assess continuous variables. A Cox proportional hazards regression model was used to identify the independent prognostic factors. Variables included in the multivariable logistic regression were age, gender, Siewert type, tumor size, histological type, pT stage, pN stage, PLN status, and adjuvant chemotherapy. The Kaplan-Meier method and the log-rank test were used to compare cumulative survival rates. All statistical analyses in this study were performed with SPSS version 26.0 (SPSS, Chicago, IL, USA) and GraphPad Prism 8.0 software (GraphPad, La Jolla, CA, USA). Nomogram and calibration curve was established by the R (x64.3.6.1) software with rms and survival packages. A value of *p* < 0.05 was regarded as statistically significant.

## Results

### Patients’ Characteristics

Three hundred cases were enrolled in this study, including 154 (51.3%) patients of Siewert type II AEG and 146 (48.7%) patients of Siewert type III AEG. The clinicopathological characteristics are summarized in **Table 1**. There were no significant differences in age, sex, ASA status, histological type, the number of dissected lymph nodes, or adjuvant chemotherapy between Siewert types II and III AEGs. Patients of Siewert type III AEG had a significantly advanced TNM stage and a larger tumor size than Siewert type II AEG. Compared with Siewert type III AEG, patients of Siewert type II AEG were more inclined to undergo a minimally invasive surgical procedure. Moreover, patients of Siewert type III AEGs showed a higher metastatic rate of lymph nodes than those with Siewert type II AEGs (7.03 ± 8.93 vs. 5.20 ± 7.07, *p* = 0.049). However, frequency of PLN metastasis was similar between Siewert types II and III AEGs (8.4% vs. 9.6%, *p* = 0.729).

**Table 1:** Clinicopathological features of the patients.

Characteristics	Type II	Type III	*p*-value
Age (years)			
<60	79 (51.3%)	71 (48.6%)	0.644
≥60	75 (48.7%)	75 (51.4%)	
Gender			
Male	122 (79.2%)	119 (81.5%)	0.619
Female	32 (20.8%)	27 (18.5%)	
ASA status			
1	99 (64.3%)	93 (63.7%)	0.955
2	40 (26.0%)	39 (26.7%)	
3	15 (9.7%)	14 (9.6%)	
BMI			
≤25	134 (87.0%)	122 (83.6%)	0.398
>25	20 (13.0%)	24 (16.4%)	
Tumor size (cm)			
≤4	107 (69.5%)	53 (36.3%)	***<0.001***
>4	47 (30.5%)	93 (63.7%)	
Histological type			
Differentiated	69 (44.8%)	55 (37.7%)	0.210
Undifferentiated	85 (55.2%)	91 (62.3%)	
Surgical approach			
Open	35 (22.7%)	55 (37.7%)	***0.009***
Laparoscopic	84 (54.6%)	71 (48.6%)	
Robotic	35 (22.7%)	20 (13.7%)	
pT stage			
T1	10 (6.5%)	1 (0.7%)	**0.037**
T2	12 (7.8%)	14 (9.6%)	
T3	20 (13.0%)	14 (9.6%)	
T4	112 (72.5%)	117 (80.1%)	
pN stage			
N0	42 (27.2%)	26 (17.8%)	***0.040***
N1	40 (26.0%)	29 (19.9%)	
N2	29 (18.8%)	43 (29.5%)	
N3	43 (28.0%)	48 (32.8%)	
TNM stage			
I	17 (11.0%)	7 (4.8%)	***0.036***
II	29 (18.8%)	19 (13.0%)	
III	108 (70.2%)	120 (82.2%)	
Number of dissected LNs	28.27 ± 11.08	27.71 ± 10.81	
<24	61 (39.6%)	57 (39.0%)	0.920
≥24	93 (60.4%)	89 (61.0%)	
PLN status			
Negative	141 (91.6%)	132 (90.4%)	0.729
Positive	13 (8.4%)	14 (9.6%)	
Adjuvant chemotherapy			
Yes	113 (73.4%)	98 (67.1%)	0.257
No	41 (26.6%)	48 (32.9%)	

Bold and italic values are statistically significant p < 0.05.

BMI, body mass index; ASA, American Society of Anesthesiologists; TNM, pathological tumor node metastasis; LNs, lymph nodes.

### Long-Term Survival

**Figure 1:**
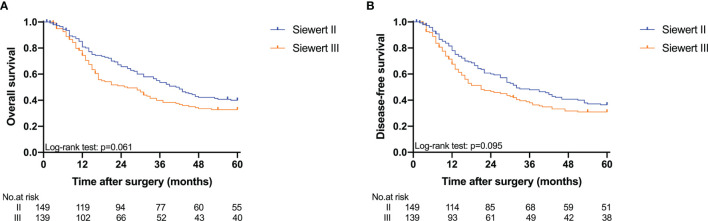
Kaplan-Meier analysis for 5-year OS and DFS between Siewert type II and III AEG. **(A)** OS; **(B)** DFS.

**Figure 2:**
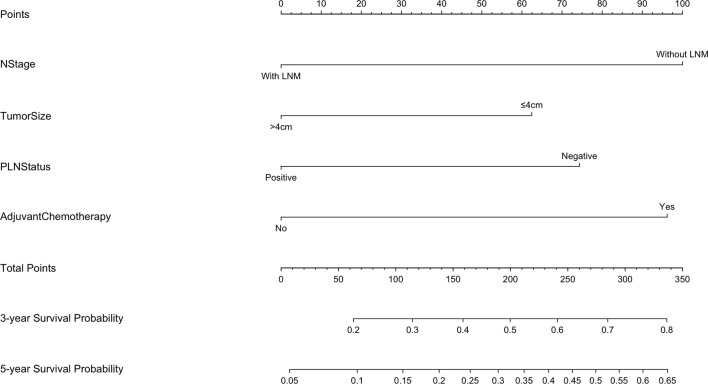
Nomogram for predicting the 3- and 5-year survival probabilities of patients with Siewert type II/III AEG (LNM, lymph node metastasis).

**Figure 3:**
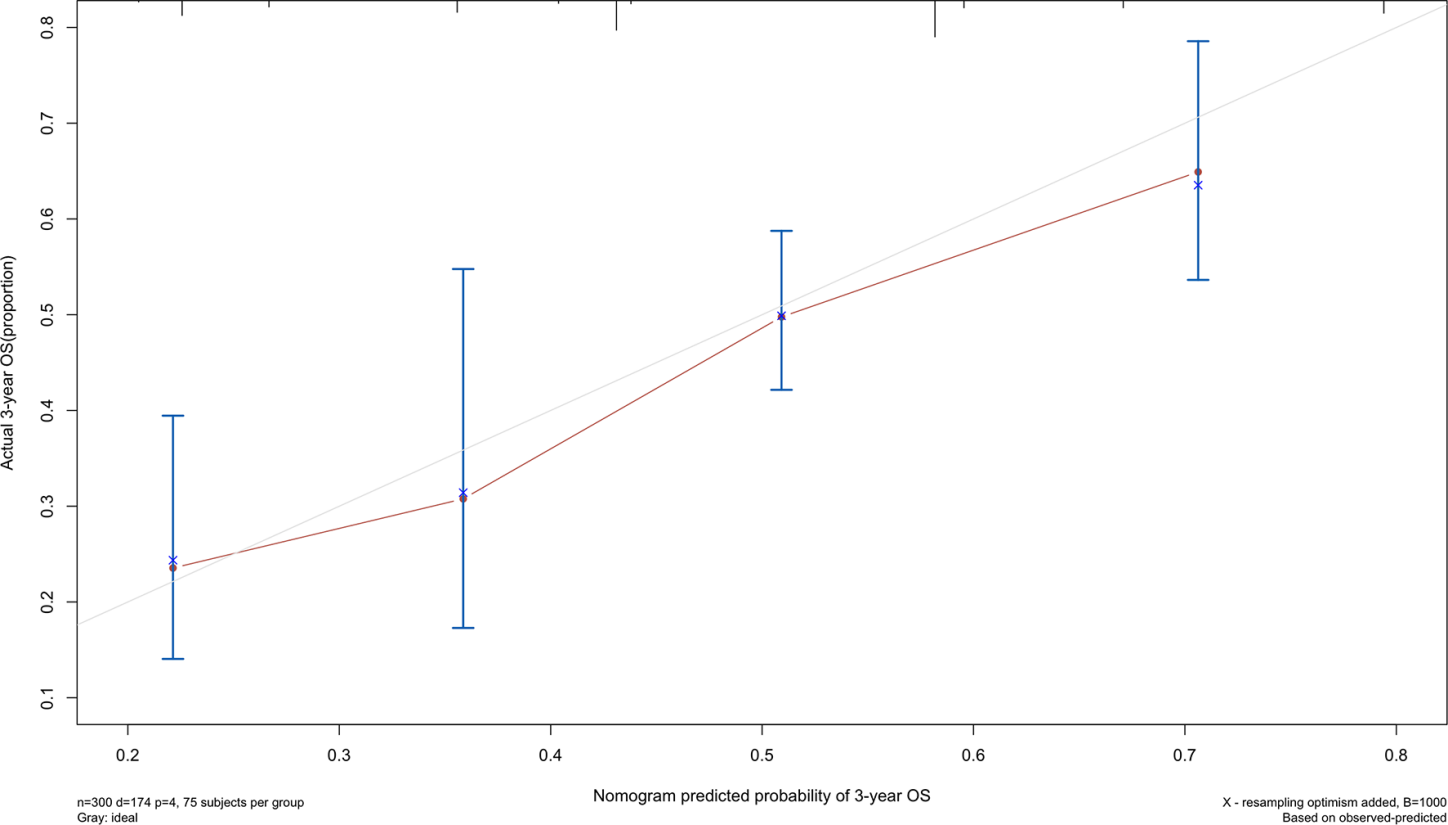
Calibration curve for nomogram of patients with Siewert type II/III AEG.

The 5-year overall survival probability of the 300 patients in this study was 36.5%. **Figure 1A** shows the 5-year OS of patients of Siewert type II and Siewert type III AEG (type II: 40.0% vs. type III: 32.8%, *p* = 0.061). Additionally, the Siewert type II and Siewert type III groups showed comparable 5-year DFS rates in **Figure 1B** (type II: 36.5% vs. type III: 30.9%, *p* = 0.095). The univariate analysis showed that tumor size, histological type, pT stage, pN stage, PLN status, and adjuvant chemotherapy were significant prognostic factors for AEGs. The Cox proportional hazards model used for the multivariate analysis demonstrated that tumor size, pN stage, and PLN status were independent risk factors for Siewert type II/III cancers, and adjuvant chemotherapy was an independent protective factor for Siewert type II/III cancers (**Table 2**). However, age, gender, Siewert type, histological type, and pT stage had nothing to do with prognostic factors. Nomogram was established by the independent prognostic factors identified in the Cox proportional hazards model for the prediction of the 3- and 5-year OS (**Figure 2**). As shown in **Figure 3**, the calibration curve demonstrated good consistency with the ideal model and the C-index of the predictive model was 0.653.

**Table 2:** Univariable and multivariable analyses of 5-year OS in Siewert type II/III patients.

	Univariable analysis			Multivariable analysis		
	HR	95% CI	*p*-value	HR	95% CI	*p*-value
Age						
<60	1.00	1.00				
≥60	0.93	0.69–1.25	0.626			
Gender						
Male	1.00	1.00				
Female	1.12	0.78–1.61	0.527			
Siewert type						
Type II	1.00	1.00				
Type III	1.34	0.99–1.80	0.053			
Tumor size						
≤4	1.00	1.00		1.00	1.00	
>4	1.60	1.19–2.15	***0.002***	1.48	1.10–2.00	***0.011***
Histological type						
Differentiated	1.00	1.00				
Undifferentiated	1.49	1.09–2.03	***0.010***			
pT stage						
T1–2	1.00	1.00				
T3–4	2.39	1.36–4.21	***0.002***			
pN stage						
N0	1.00	1.00		1.00	1.00	
N1–3	1.96	1.31–2.92	***0.001***	1.89	1.25–2.84	***0.002***
PLN status						
Negative	1.00	1.00		1.00	1.00	
Positive	2.01	1.28–3.14	***0.002***	1.59	1.01–2.51	***0.046***
Adjuvant chemotherapy						
No	1.00	1.00		1.00	1.00	
Yes	0.571	0.42–0.79	***0.001***	0.55	0.40–0.76	***<0.001***

Bold and italic values are statistically significant p < 0.05.

HR, hazard ratio; CI, confidence interval.

**Figure 4:**
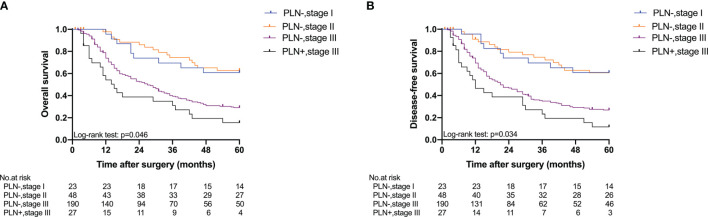
Five-year OS and DFS of all patients classified by TNM stage and PLN status. **(A)** OS; **(B)** DFS (PLN−, stage III vs. PLN+, stage III, *p* = 0.046 for OS, *p* = 0.034 for DFS).

**Figure 5:**
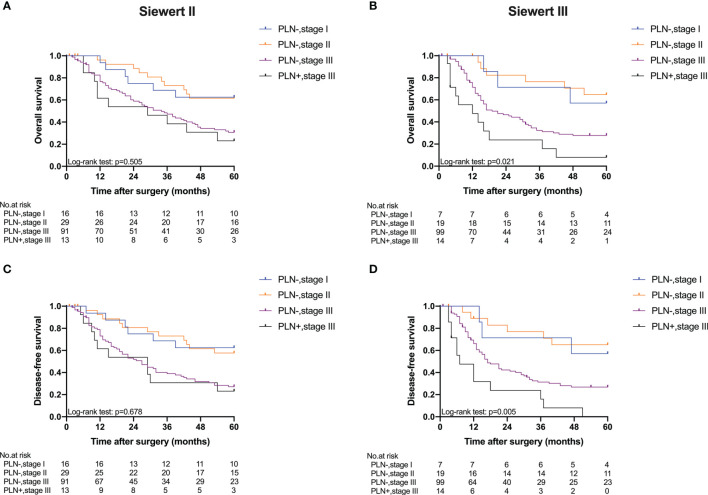
Five-year OS and DFS of Siewert type II and III AEGs classified by TNM stage and PLN status. **(A)** OS of Siewert type II, *p* = 0.505. **(B)** OS of Siewert type III, *p* = 0.021. **(C)** DFS of Siewert type II, *p* = 0.678. **(D)** DFS of Siewert type III, *p* = 0.005 (PLN−, stage III vs. PLN+, stage III).

Five-year OS and 5-year DFS curves of patients with Siewert type II/III cancers classified by TNM stage and PLN status are shown in **Figure 4**. In the current study, all PLN-positive patients of Siewert type II/III AEG were diagnosed with stage III disease. Of the patients in the Siewert type II/III AEG group, the PLN-positive patients with stage III AEG had a significantly worse 5-year OS and 5-year DFS than PLN-negative patients with stage III AEG (15.5% vs. 29.1%, *p* = 0.046; 11.6% vs. 26.9%, *p* = 0.034, respectively). When the subgroup analysis was performed for different Siewert types (**Figure 5**), patients with stage III PLN-positive cancer had a comparable prognosis in terms of 5-year OS and 5-year DFS to those with stage III PLN-negative patients in the Siewert type II AEG group (23.1% vs. 30.6%, *p* = 0.505; 23.1% vs. 27.1%, *p* = 0.678, respectively). However, of those in the Siewert type III AEG group, patients with stage III PLN-positive cancer had a significantly worse 5-year OS and 5-year DFS than those with stage III cancer without PLN metastasis (7.9% vs. 27.8%, *p* = 0.021; 0 vs. 26.8%, *p* = 0.005; respectively).

**Figure 6:**
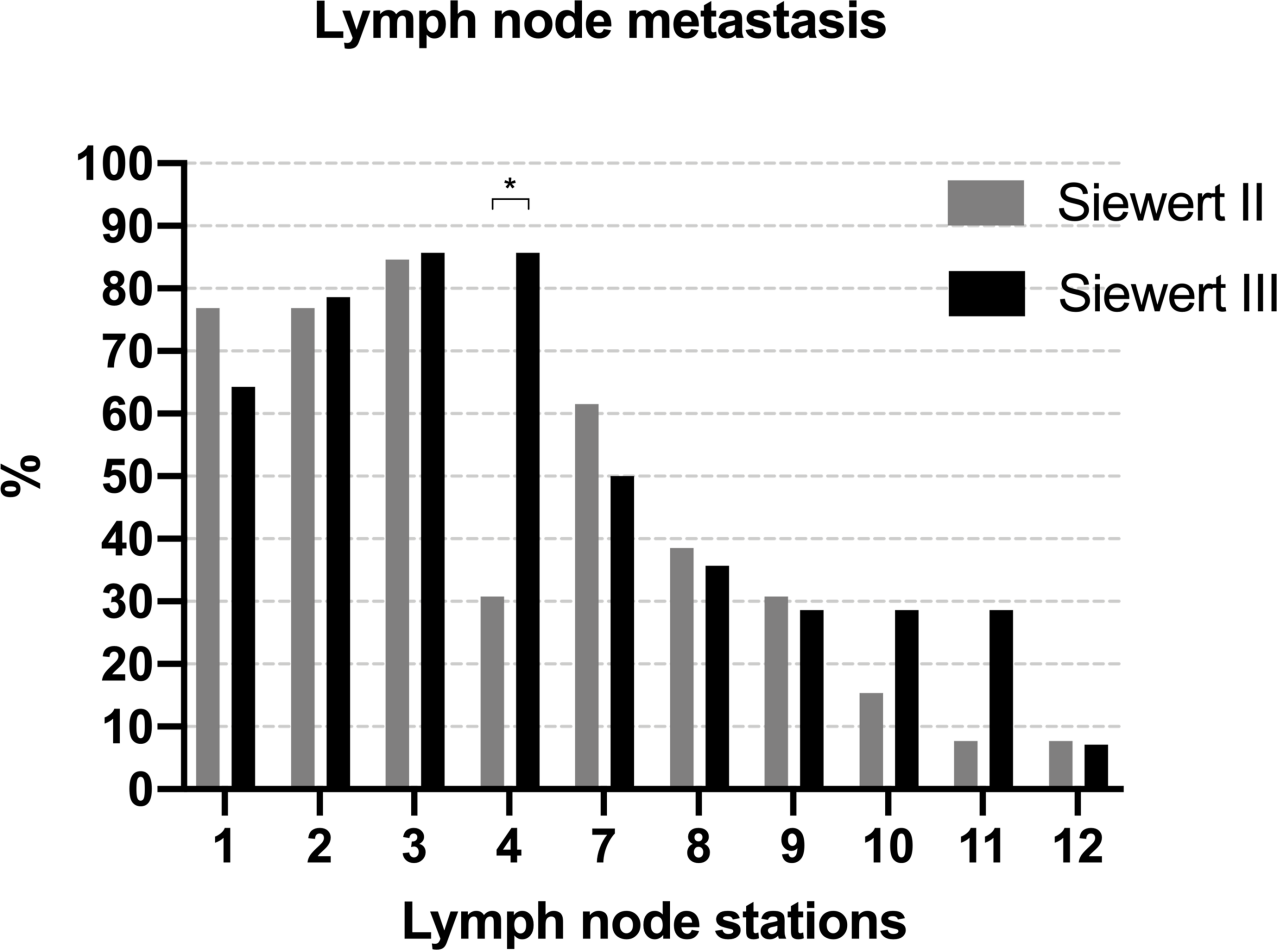
The metastatic rate of lymph node in PLN-positive Siewert type II/III cancers at each station (^*^ *p* = 0.004).

**Figure 6** summarizes the metastatic rate of the lymph nodes in PLN-positive Siewert type II/III cancers at each station. Lymph node metastasis was more frequent in lymph nodes No. 1–3, 7, 8, and 9 in both groups. Patients of Siewert type II AEG showed the lower metastatic rate of lymph node No. 4 than in Siewert type III AEG (30.8% vs. 85.7%, *p* = 0.004). A trend toward a lower metastatic frequency of the No. 10 and 11 lymph nodes was seen in Siewert type II AEG compared with Siewert type III AEG (No. 10, 15.4% vs. 28.6%, *p* = 0.648; No. 11, 7.7% vs. 28.6%, *p* = 0.326).

### Calculated Index for Each Lymph Node

According to the IEBLDs, the dissection of lymph nodes 1, 2, 3, 7, 8, 9, and 11 come up with an excellent outcome (IEBLD >3.0) in both groups (**Table 3**). In contrast, the IEBLDs of stations located far from the EGJ were relatively low. The IEBLD was less than 3.0 in stations 4, 5, 6, and 12 in patients of Siewert type II/III AEGs. In Siewert type III AEG, the IEBLD of lymph node No. 10 showed a high therapeutic benefit.

**Table 3:** Therapeutic value index of each lymph node station in Siewert type II/III patients.

Station	No. of LNM		No. of LND		Metastatic incidence (%)		5-year survival rate (%)		IEBLD	
	Type II	Type III	Type II	Type III	Type II	Type III	Type II	Type III	Type II	Type III
No. 1	61	65	147	133	41.5%	48.9%	19.7%	21.5%	8.2	10.5
No. 2	52	69	143	132	36.4%	52.3%	21.2%	23.2%	7.7	12.1
No. 3	73	89	153	133	47.7%	66.9%	31.5%	23.6%	15.0	15.8
No. 4	12	27	149	134	8.1%	20.1%	16.7%	11.1%	1.4	2.2
No. 5	8	9	80	86	10%	10.5%	25%	11.1%	2.5	1.2
No. 6	9	9	120	112	7.5%	8.0%	22.2%	11.1%	1.7	0.9
No. 7	52	58	138	126	37.7%	46.0%	25%	25.9%	9.4	11.9
No. 8	28	25	134	118	20.9%	21.2%	17.9%	24%	3.7	5.1
No. 9	18	19	98	95	18.4%	20%	22.2%	21.1%	4.1	4.2
No. 10	6	12	73	64	8.2%	18.8%	0	16.7%	0	3.1
No. 11	14	16	87	77	16.1%	20.8%	21.4%	18.8%	3.4	3.9
No. 12	4	4	50	34	8%	11.8%	25%	0	2	0

IEBLD, index of estimated benefit from lymph node dissection; No. of LNM, number of patients with lymph node metastasis; No. of LND, number of patients in whom each lymph node station was dissected.

**Tables 4** and **5** show the IEBLDs based on the tumor diameter (≤4 and >4 cm) in Siewert type II AEG and Siewert type III AEG, respectively. The index values for lymph nodes No. 1, 2, 3, 7, 8, 9, and 11 were still high regardless of tumor diameter for Siewert type II/III AEGs. In contrast, the IEBLDs of lymph nodes No. 4, 10, and 12 in Siewert type II AEG and the No. 12 lymph node in Siewert type III AEG were still less than 3.0 regardless of tumor diameter.

**Table 4:** Therapeutic value index of each lymph node station in Siewert type II AEGs based on tumor diameter.

Station	No. of LNM		No. of LND		Metastatic incidence (%)		5-year survival rate (%)		IEBLD	
	TD ≤4	TD >4	TD ≤4	TD >4	TD ≤4	TD >4	TD ≤4	TD >4	TD ≤4	TD >4
No. 1	36	25	102	45	35.3%	55.6%	25%	12%	8.8	6.7
No. 2	27	25	98	45	27.6%	55.6%	22.2%	20%	6.1	11.1
No. 3	45	28	106	47	42.5%	59.6%	40%	17.9%	17	10.7
No. 4	9	3	105	44	8.6%	6.8%	11.1%	33.3%	1	2.3
No. 5	5	3	58	22	8.6%	13.6%	20%	33.3%	1.7	4.5
No. 6	5	4	88	32	5.7%	12.5%	20%	25%	1.1	3.1
No. 7	29	23	98	40	29.6%	57.5%	31.0%	17.4%	9.2	10.0
No. 8	14	14	94	40	14.9%	35%	21.4%	14.3%	3.2	5.0
No. 9	11	7	68	30	16.2%	23.3%	27.3%	14.3%	4.4	3.3
No. 10	4	2	51	22	7.8%	9.1%	0	0	0	0
No. 11	9	5	54	33	16.7%	15.2%	22.2%	20%	3.7	3
No. 12	3	1	39	11	7.7%	9.1%	33.3%	0	2.6	0

IEBLD, index of estimated benefit from lymph node dissection; No. of LNM, number of patients with lymph node metastasis; No. of LND, number of patients in whom each lymph node station was dissected.

**Table 5:** Therapeutic value index of each lymph node station in Siewert type III AEGs based on tumor diameter.

Station	No. of LNM		No. of LND		Metastatic incidence (%)		5-year survival rate (%)		IEBLD	
	TD ≤4	TD >4	TD ≤4	TD >4	TD ≤4	TD >4	TD ≤4	TD >4	TD ≤4	TD >4
No. 1	26	39	50	83	52%	47.0%	15.4%	25.6%	8.0	12.0
No. 2	23	46	49	83	46.9%	55.4%	21.7%	23.9%	10.2	13.2
No. 3	34	55	51	82	66.7%	67.1%	20.6%	25.5%	13.7	17.1
No. 4	5	22	51	83	9.8%	26.5%	0	13.6%	0	3.6
No. 5	0	9	30	56	0	16.1%	NA	11.1%	NA	1.8
No. 6	1	8	42	70	2.4%	11.4%	0	12.5%	0	1.4
No. 7	21	37	49	77	42.9%	48.1%	28.6%	24.3%	12.3	11.7
No. 8	9	16	46	72	19.6%	22.2%	22.2%	25%	4.4	5.6
No. 9	8	11	40	55	20%	20%	25%	18.2%	5	3.6
No. 10	2	10	26	38	7.7%	26.3%	0	20%	0	5.3
No. 11	3	13	24	53	12.5%	24.5%	33.3%	15.4%	4.2	3.8
No. 12	1	3	16	18	6.2%	16.7%	0	0	0	0

IEBLD, index of estimated benefit from lymph node dissection; No. of LNM, number of patients with lymph node metastasis; No. of LND: Number of patients in whom each lymph node station was dissected.

Interestingly, the IEBLDs of lymph nodes No. 5 and 6 revealed high therapeutic efficacy of dissection in Siewert type II AEG with a tumor diameter >4 cm. However, the IEBLDs of lymph nodes No. 5 and 6 were still low regardless of tumor diameter in Siewert type III AEG. Similarly, the index values for stations No. 4 and 10 were high in Siewert type III AEG in which the tumor diameter was >4 cm.

## Discussion

In the current study, the dissection of abdominal stations 1, 2, 3, 7, 8, 9, and 11 are supposed to be a routine procedure for Siewert type II/III AEG. Additionally, in Siewert type III AEG, the IEBLD of lymph node No. 10 showed a high therapeutic benefit of dissection. In contrast, dissection of the No. 4, 5, 6, and 12 lymph nodes is not recommended because of the relatively low IEBLDs in patients of Siewert type II/III AEG. Previous studies demonstrated the highest IEBLD of lymph nodes 1, 2, 3, and 7 as well as the lowest IEBLD of lymph nodes 5, 6, and 12 in Siewert type II/III AEG (4, 22). In this study, a subgroup analysis by tumor diameter showed that the IEBLDs of lymph nodes No. 5 and 6 revealed high therapeutic efficacy of dissection in Siewert type II AEG with a tumor diameter >4 cm and led to a recommendation for dissection of PLNs in Siewert type II AEG with a tumor diameter >4 cm. As far as we are concerned, no study has recommended PLN dissection based on IEBLDs for Siewert type II AEG with a tumor diameter >4 cm before. To date, several large randomized clinical trials have demonstrated that transabdominal gastrectomy with resection of the lower esophagus should be the standard treatment strategy for Siewert type II/III AEGs with esophageal invasion less than 3 cm, but the extent of optimal gastric resection remains unclear (20, 23, 24). One of the obvious advantages of total gastrectomy is to achieve a more thorough lymphadenectomy. Proximal gastrectomy is known as function-preserving and achieves a survival rate similar to that of total gastrectomy (25). Nevertheless, proximal gastrectomy for AEG has not been widely accepted because its nutritional benefits remain uncertain with a high risk of reflux symptoms. The No. 5 and No. 6 lymph nodes are not dissected in proximal gastrectomy.

The dissection of PLNs is controversial due to its uncertain potential benefit for Siewert type II/III AEGs. Several reports from Japan have shown that the metastatic rate of No. 5 or 6 lymph nodes in Siewert type II/III patients is very low (20, 22). Recently, one study has revealed that the incidence of PLN metastasis was less than 1% and concluded that PLN dissection offered marginal therapeutic benefits for patients with AEG despite their high dissection rates (8). However, in that study, most patients were in the early stages of the disease, and cases in the T1 and T2 stages accounted for 78.1% (1,861/2,384); moreover, patients with AEG were classified based on the Nishi classification. In accordance with the studies mentioned above, the JGCA states that it is not necessary to dissect PLNs for AEGs with a tumor diameter ≤4 cm (7). However, the guidelines do not mention relevant recommendations for AEG with a tumor diameter >4 cm. Therefore, in the present study, we have chosen diameter of 4 cm as the threshold to investigate the metastasis rate of PLNs. In Japan, patients diagnosed with AEG are mostly at an early stage, whereas approximately 80% of patients are diagnosed at advanced stages, and cases of AEG with a tumor diameter >4 cm account for a large proportion in China (12, 13). Chen et al. and Hu et al. revealed that the frequency of PLN metastasis exceeded 10% in AEG (9, 10). Similarly, in the current study, the incidence of PLN metastasis in the total cohort was 9%. One possible explanation for the discrepancy among these reports is that patients with pT4 account for 70% of those in studies that report a high incidence of PLN metastasis. Recently, a prospective nationwide multicenter study revealed that the metastasis rate of PLNs was more than 10% if the tumor size exceeded 6 cm (11). Therefore, whether diameter is a risk factor that increases the incidence of PLN metastasis in Siewert type II/III AEGs is worthy of further investigation. Similarly, our reports showed that the incidence of PLN metastasis exceeded 10% when the tumor diameter was >4 cm in Siewert type II/III AEGs. Surprisingly, our results showed that the IEBLDs of lymph nodes 5 and 6 yielded high therapeutic efficacy in Siewert type II AEG with a tumor diameter >4 cm. However, in Siewert type III AEG, the IEBLDs of lymph nodes 5 and 6 were still low regardless of tumor diameter.

The JGCA comes up with a flow diagram for the identification of the extent of the lymphadenectomy for AEG with a tumor diameter ≤4 cm. However, these reports do not present a detailed investigation of the IEBLDs based on tumor diameter. For all we know, we are the first to evaluate the IEBLDs of Siewert type II/III AEGs based on tumor diameter. Our results showed that the IEBLDs of lymph nodes 1, 2, 3, 7, 8, 9, and 11 were still high, which indicates that dissection of these nodes is inevitable regardless of tumor diameter in Siewert type II/III AEGs. We also found high IEBLDs in greater curvature and splenic hilar lymph nodes in Siewert type III AEG in which the tumor diameter was >4 cm, which was consistent with the study by Huang et al., who proposed that spleen-preserving No.10 lymphadenectomy is associated with better prognosis of Siewert type III AEG with a tumor diameter >4 cm (26). This suggests that No. 10 lymphadenectomy is recommended for Siewert type III AEG with a tumor diameter >4 cm (26).

Although Siewert type III cancers had more metastatic lymph nodes in this study, the metastasis rate of PLNs was comparable between Siewert type II and Siewert type III AEGs. This finding is consistent with that of Huang et al., who suggested that the reason might be the limited sample of PLN-positive patients (17). In the present study, tumor size, pN stage, and PLN status were independent risk factors for OS in Siewert type II/III AEGs. These results reflect those of Huang et al., who also found that age, tumor size, pT stage, pN stage, pM stage, PLN status, and the number of positive lymph nodes were related to poorer OS (17). These findings were also reported by Wu et al., who revealed that pT stage, lymph node ratio, and PLN status have something to do with OS (27). Then, we further investigated the prognosis of PLN-positive Siewert type II/III AEGs. Of those with Siewert type II/III AEG, PLN-positive patients with stage III AEG had a significantly worse prognosis than PLN-negative patients with stage III AEG. Of those with Siewert type II AEG, a subgroup analysis by Siewert type showed that PLN-positive patients with stage III AEG had a similar prognosis to PLN-negative patients with stage III AEG. However, in the Siewert type III AEG group, PLN-positive patients with stage III AEG had a significantly worse prognosis than PLN-negative patients with stage III AEG. However, the findings of the present study do not support the previous research. Huang et al. proposed that the survival of PLN-positive patients was poorer than that of stage III patients without PLN metastasis regardless of Siewert type (17). To investigate the reason why the prognosis of PLN-positive patients of Siewert type II AEG is better than that of patients with Siewert type III AEG, we assessed the metastatic rate of the lymph nodes in PLN-positive Siewert type II/III cancers at each station (**Figure 6**). We found that PLN-positive Siewert type III AEGs had a higher probability of lymph node metastasis on the greater curvature of the stomach and a relatively wider range of metastatic lymph nodes than PLN-positive Siewert type II AEGs, which may lead to a worse prognosis and may partly explain such a discrepancy. Another explanation for this result may be that dissection of PLNs in Siewert type II AEG with a tumor diameter >4 cm yields a high therapeutic benefit based on previous IEBLD results and improves survival.

In Siewert type II AEG with a tumor diameter ≤4 cm, the IEBLDs of PLNs were low in the current study. A previous study demonstrated that lymph node metastasis is more likely to occur in Siewert type II AEGs with larger tumor diameters (20). Therefore, we suggest that dissection of PLNs may be unnecessary and that the distal stomach could be preserved in Siewert type II AEG with a tumor diameter ≤4 cm. Further researches are required to explore the underlying benefits of proximal gastrectomy over total gastrectomy, such as a better postoperative quality of life (28). However, based on the IEBLDs of PLNs (>3.0), we suggested the adoption of total gastrectomy with lymphadenectomy for Siewert type II AEG with a tumor diameter >4 cm. The current study showed that the IEBLDs of PLNs were low in Siewert type III AEG regardless of tumor diameter. A previous study revealed that lymphatic flow originates from the middle third of the stomach to the lower perigastric lymph nodes (29). Typically, Siewert type III AEG always has a large tumor diameter and therefore invades the middle third of the stomach. Hence, total gastrectomy with D2 lymphadenectomy is suitable for Siewert type III AEG regardless of tumor size.

There were several limitations of this study. First of all, it was retrospective research performed at a single institution, and not all of the patients received a uniform extent of lymphadenectomy. We excluded patients with retrieved lymph nodes <16 for the minimization of false-negative influence on the current results. Second, in this study, the value of mediastinal lymph node dissection was not assessed because mediastinal lymph node dissection was not a routine procedure, especially in the early study period. The 14th edition of the JGCA guidelines recommended lower mediastinal lymph node dissection for patients with AEG (18), which is not mentioned in the former JGCA guidelines during the early study period. Third, to accurately evaluate the histological status of each lymph node, we excluded cases who received neoadjuvant chemotherapy which may include a selection bias. The present results require more accurate evidence from a large multicenter randomized study.

## Conclusion

We recommended that a total gastrectomy with lymphadenectomy should be adopted for Siewert type II AEG with a tumor diameter >4 cm and Siewert type III AEG. In contrast, omitting dissection of PLNs and preservation of the distal part of the stomach might be optional for patients of Siewert type II AEG with a tumor diameter ≤4 cm.

## Data Availability Statement

The original contributions presented in the study are included in the article/supplementary material. Further inquiries can be directed to the corresponding author.

## Ethics Statement

All procedures followed were in accordance with the ethical standards of the responsible committee on human experimentation (Ethics Committee of the First Affiliated Hospital of Army Medical University, PLA, China, approval number: KY2020272) and with the Helsinki Declaration of 1964 and later versions. Informed consent or substitute for it was obtained from all patients for being included in the study.

## Author Contributions

XL: contributed to study design, data collection, and writing. ZL: contributed to data collection. CT: contributed to data collection. XY: contributed to data collection. JX: contributed to data collection. JL: contributed to data collection. AM: contributed to data collection. YS: contributed to data analysis. FQ: contributed to data analysis. PY: contributed to data analysis. YZ: contributed to study design. All authors contributed to the article and approved the submitted version.

## Funding

This research is funded by the National Natural Science Foundation of China (Grant Number: 81872016).

## Conflict of Interest

The authors declare that the research was conducted in the absence of any commercial or financial relationships that could be construed as a potential conflict of interest.

## Publisher’s Note

All claims expressed in this article are solely those of the authors and do not necessarily represent those of their affiliated organizations, or those of the publisher, the editors and the reviewers. Any product that may be evaluated in this article, or claim that may be made by its manufacturer, is not guaranteed or endorsed by the publisher.
